# Genome-wide transcriptomics analysis of genes regulated by GATA4, 5 and 6 during cardiomyogenesis in *Xenopus laevis*

**DOI:** 10.1016/j.dib.2018.01.005

**Published:** 2018-01-17

**Authors:** Boni A. Afouda, Adam T. Lynch, Eduardo de Paiva Alves, Stefan Hoppler

**Affiliations:** a Institute of Medical Sciences, Foresterhill Health Campus, University of Aberdeen, Scotland, UK; b Centre for Genome-Enabled Biology and Medicine, King's College Campus, University of Aberdeen, Scotland, UK

## Abstract

The transcription factors GATA4, GATA5 and GATA6 play important roles in heart muscle differentiation. The data presented in this article are related to the research article entitled “Genome-wide transcriptomics analysis identifies sox7 and sox18 as specifically regulated by gata4 in cardiomyogenesis” (Afouda et al., 2017) [1]. The present study identifies genes regulated by these individual cardiogenic GATA factors using genome-wide transcriptomics analysis. We have presented genes that are specifically regulated by each of them, as well those regulated by either of them. The gene ontology terms (GO) associated with the genes differentially affected are also presented. The data set will allow further investigations on the gene regulatory network downstream of individual cardiogenic GATA factors during cardiac muscle formation.

**Specifications Table**

**Table:** 

Subject area	Biology
More specific subject area	Molecular Biology
Type of data	Tables
How data was acquired	Experimental samples were prepared as described in the related research article [1]. Illumina TruSeq RNA libraries were constructed and sequenced with Illumina HiSeq_2500. 100bp paired-end sequencing reads were aligned to *Xenopus laevis* genome (version 9.1) using HiSat2. Quantification was done using featureCounts and differential expression was performed using DESeq2 with an adjusted p value <0.05. Identification of differentially expressed genes was done using a threshold of log-2 fold change (>1 for at least two times increase or <-1 for at least two times reduced) in comparison to control. Analyses of differentially expressed genes was done using Partek genomics Suite 6.6.
Data format	Filtered and analysed
Experimental factors	Cardiogenic explant samples (control) versus GATA4, 5 and 6 depleted samples. Each sample contain at least three biological replicates and each replicates are pooled of 30 explants.
Experimental features	Total RNA was extracted from Activin-injected animal cap (cardiogenic explants use as control) as well as GATA4, 5 and 6 depleted explants collected at developmental stage 32 (NieuwKoop Faber).
Data source location	*Xenopus laevis* are Lab-bred and sourced from NASCO (Fort Atkinson, Wisconsin, USA) and kept at the Institute of Medical Sciences Animal Research Unit (Foresterhill Health Campus, Aberdeen- Scotland).
Data accessibility	Data are with this article

**Value of the data**

• GATA4, 5 and 6 are important regulators of heart muscle differentiation (cardiomyogenesis).
• These data identify genes differentially regulated by each of these GATA factors during cardiomyogenesis.
• These data also identify genes that are regulated by these factors in common during this process.
• These data will help us understand the molecular mechanisms that govern the function of these factors and therefore improve our knowledge about the gene regulatory network involved in cardiomyogenesis.
• The analysis of gene ontology (GO) terms associated with cardiogenic GATA-regulated genes provides the different biological processes in which these factors are involved in during normal development and homeostasis.

## 1 Data

The data represents RNA-Seq performed on cardiogenic samples, single GATA4, 5 and 6 depleted samples as well as samples with GATA4, 5 and 6 triple depleted, described in the related research article [1].

**Fig. 1:**
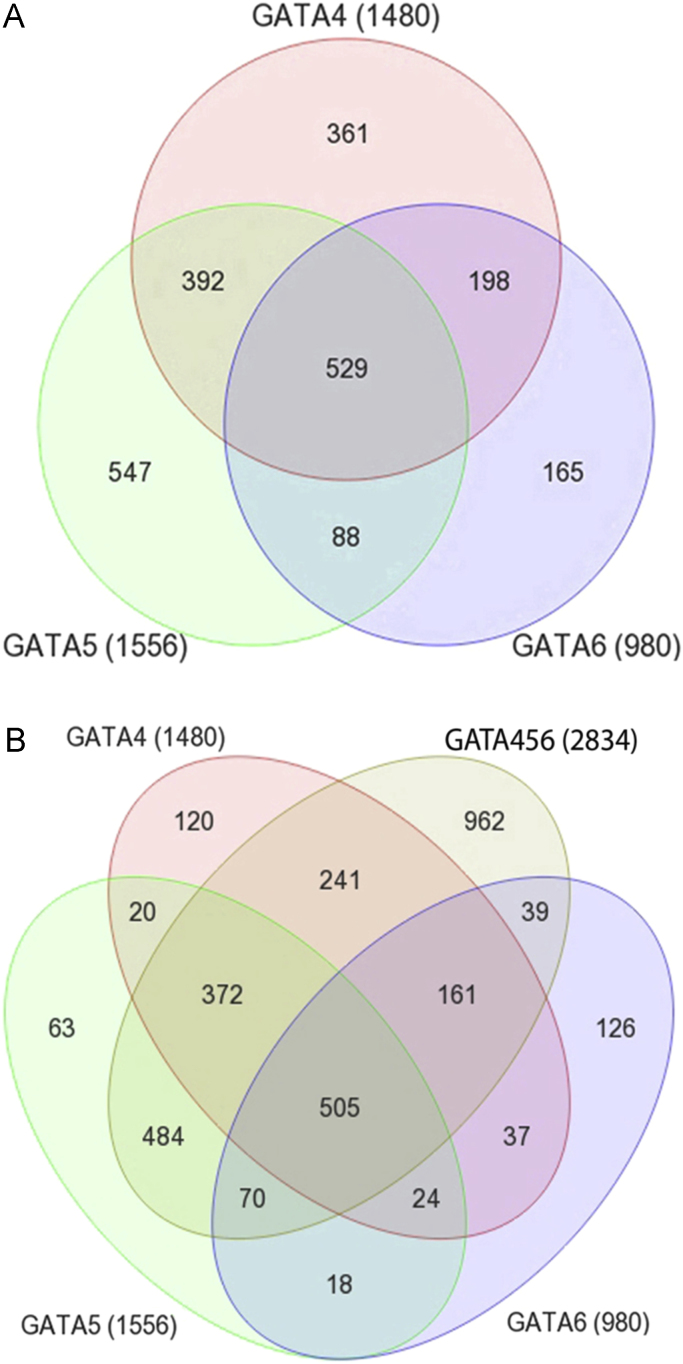
Venn diagrams of numbers of genes that are differentially expressed compared to Activin-injected control with at least a two-fold reduction in expression and an adjusted p value of <0.05. A: gata-dependent genes with decreased expression in single gata4, gata5 or gata6 knockdown, respectively (for list of genes, see Table 1). B: gata-dependent genes with decreased expression in triple gata4, gata5 or gata6 knockdown compared to those with decreased expression in single gata4, gata5 and gata6 knockdown, respectively (for list of genes, see Table 2).

In Table 1 (sheets 1–4), Sheet 1 represents the list of 529 shared genes with significantly reduced expression in the single knockdown (KD) of either *gata4*, *gata5* or *gata6* (see Fig. 1A). Sheet 2 represents the list of 361 genes that are specifically reduced only by knockdown of *gata4* (see Fig. 1A). Sheet 3 represents the list of 547 genes that are exclusively reduced by knockdown of *gata5* (see Fig. 1A). Sheet 4 represents the list of 165 genes that are specifically reduced by knockdown of *gata6* (see Fig. 1A). Ifc, log2 fold change.

In Table 2 (sheets 1–3), Sheet 1 represents the list of 2835 genes with significantly reduced expression in the triple knockdown of *gata4*, *gata5* and *gata6* (see Fig. 1B). Sheet 2 represents the list of 505 shared genes that are reduced by single knockdown of either *gata4*, *gata5* or *gata6* or the triple knockdown (see Fig. 1B, they represent a subset of the 529 genes shown in Table 1, sheet 1) and sheet 3 represents list of 241 shared genes that are specifically reduced by *gata4* single knockdown and in the triple knockdown (see Fig. 1B, they represent a subset of the 361 genes shown in Table 1, sheet 2).

In Table 3 (sheets 1–4), Sheet 1: The 1480 genes with statistically reduced expression in the *gata4* knockdown (see Fig. 1A) have been subjected to gene ontology (GO) analysis. Among the GO terms enriched and presented in sheet 3 is the GO class “Regulation of transcription”. List of genes within “regulation of transcription” GO term including *sox7* and *sox18* (highlighted in red). Sheet 2: The 361 genes that are exclusively reduced by *gata4* knockdown (see Fig. 1A) have been subjected to GO analysis. Among the GO terms enriched and presented in sheet 4 is the GO class “DNA-binding”. List of genes within “DNA-binding” GO term including *sox7* and *sox18* (highlighted in red). Sheet 3: represents GO biological process terms associated with all the 1480 genes that are reduced in *gata4* knockdown (see Fig. 1A). Sheet 4: represents GO biological processes associated with the 361 genes that are exclusively reduced upon *gata4* knockdown (see Fig. 1A).

## 2 Experimental design, materials and methods

### 2.1 Expression constructs, mRNA synthesis and morpholinos

Activin β B DNA constructs for mRNA synthesis have been described previously [2], [3], [4]. All fusion plasmids were *Sal*I-linearized and in vitro transcribed with SP6 using mMESSAGE mMACHINE kits (Ambion) according to the manufacturer's instruction. 50fg of RNA for Activin were injected. Xenopus gata4 splice morpholino, gata5 splice morpholino [5], gata6 morpholino [6], Xsox7 and Xsox18 [7] morpholinos have been previously described. The amounts of MOs injected per embryo are: 50ng (gata4, 4MO), 8ng (gata5, 5MO), 10ng (gata6, 6MO), 30ng for single (sox7, 7MO) and (sox18, 18MO) and 15ng of each when combined.

### 2.2 Embryos and explants culture, experimental sample production, RNA extraction

*Xenopus laevis* embryos were obtained as previously described [2]. Embryos and explants culture as well as embryos injection were as previously described [3], [4]. Animal cap explants were excised as previously described [8]. Experimental samples were prepared and validated as described in the related research article [1].

### 2.3 RNA-seq experiments and analysis

At least 30 explants were used for RNA preparation with a previously described protocol [3], [4]. RNA quantity and quality were checked on electrophoretic agarose gel, a fraction of which was used for validation with gene expression analysis by quantitative RT-PCR [2] to confirm expected increase or decrease of known control gene expression before RNA-seq sequencing. RNA was isolated from three independent biological replicates for each condition. Illumina TruSeq RNA libraries were constructed and sequenced on the Illumina HiSeq_2500 platform at the Earlham Institute, Norwich Research Park, Norwich, UK. 100 bp paired-end sequencing reads were aligned to the *Xenopus laevis* genome (version 9.1) using HiSat2 [9] and quantification was done using featureCounts [10]. Differential expression analysis was performed using DESeq. 2 [11] with an adjusted p value <0.05. Differentially expressed genes were identified using a threshold of log-2 fold change > 1 (for at least two times increased) or <-1 (for at least two times reduced) in comparison to Activin-induced Xenopus animal cap cardiac explant controls. Analyses of differentially expressed genes were performed using Partek genomics Suite 6.6. For gene ontology (GO) analyses, GO classes containing at least six genes were taken into consideration.

## Appendix A Supplementary material

Table 1 Redundancy and specific requirements of gata4, 5 and 6 during cardiomyogenesis.

Table 2 Specific and redundant requirements of gata4 during cardiomyogenesis.

Table 3 sox7 and sox18 are among transcription factor genes that require gata4 function for expression during cardiomyogenesis.

## Footnotes

Appendix A Supplementary data associated with this article can be found in the online version at doi:10.1016/j.dib.2018.01.005.
